# The Carnot Cycle, Reversibility and Entropy

**DOI:** 10.3390/e23070810

**Published:** 2021-06-25

**Authors:** David Sands

**Affiliations:** Department of Physics and Mathematics, University of Hull, Hull HU6 7RX, UK; david.sands@physicseducationinternational.com

**Keywords:** Carnot cycle, entropy, reversibility, irreversibility, Clausius

## Abstract

The Carnot cycle and the attendant notions of reversibility and entropy are examined. It is shown how the modern view of these concepts still corresponds to the ideas Clausius laid down in the nineteenth century. As such, they reflect the outmoded idea, current at the time, that heat is motion. It is shown how this view of heat led Clausius to develop the entropy of a body based on the work that could be performed in a reversible process rather than the work that is actually performed in an irreversible process. In consequence, Clausius built into entropy a conflict with energy conservation, which is concerned with actual changes in energy. In this paper, reversibility and irreversibility are investigated by means of a macroscopic formulation of internal mechanisms of damping based on rate equations for the distribution of energy within a gas. It is shown that work processes involving a step change in external pressure, however small, are intrinsically irreversible. However, under idealised conditions of zero damping the gas inside a piston expands and traces out a trajectory through the space of equilibrium states. Therefore, the entropy change due to heat flow from the reservoir matches the entropy change of the equilibrium states. This trajectory can be traced out in reverse as the piston reverses direction, but if the external conditions are adjusted appropriately, the gas can be made to trace out a Carnot cycle in P-V space. The cycle is dynamic as opposed to quasi-static as the piston has kinetic energy equal in difference to the work performed internally and externally.

## 1. Introduction

The Carnot cycle is the foundation upon which modern thermodynamics has been built, but we owe our modern understanding not to Carnot but to Clausius. Carnot had framed his theory of the heat engine in terms of caloric, the invisible, indestructible material that was believed to be heat, but Clausius re-worked Carnot’s ideas to take into account not only the notion then emerging that heat was a form of motion but also the idea that heat and work were equivalent [1]. Although later developments by Caratheodory, aimed at putting thermodynamics on a more mathematically sound footing, and the development of irreversible thermodynamics in the second half of the twentieth century, would seem to have reduced the dependence on the foundations laid down by Clausius, the modern notions of reversibility and entropy are still those laid down by Clausius in the nineteenth century. As Planck made clear in 1926 [2], even the commonly quoted form of the second law is due to Clausius: there is a property of thermodynamic systems called entropy that is either constant or increases in all changes of the system.

This is not the original form in which Clausius expressed the second law: heat cannot pass from a colder to a hotter body without some other effect occurring at the same time. In fact, it is not clear that these two forms are equivalent, whereas Clausius’s original form is equivalent to Kelvin’s statement: it is impossible to convert an amount of heat completely into work in a cyclic process in the absence of other effects. The cyclic process is central to both Kelvin’s and Clausius’s original statements because they were formulated with reference to heat engines. Indeed, Zemansky uses two ideal Carnot engines working in tandem, one operating as a heat engine and the other as a refrigerator, to show that the two are equivalent [3]. How, then, do we make the transition from laws developed with reference to cyclic processes to a form of the law applicable to non-cyclic processes?

The answer lies in Clausius’s Sixth Memoir, published originally in 1862. This is one of nine papers by Clausius on thermodynamics originally published between 1850 and 1865 that were published together in one volume in 1867 [4]. Each paper is referred to as a memoir and the whole collection describes the evolution of his thinking from his re-working of Carnot’s theory to his development of entropy. It is in the Sixth Memoir that he describes his thinking about the extension of the second law to non-cyclic processes and the function he later came to name as entropy. It is not intended here to present a detailed historical analysis, but to give an insight into the origins of Clausius’s conceptions of both entropy and reversibility prior to presenting an alternative view of the Carnot cycle.

There is still no generally accepted physical meaning attached to thermodynamic entropy, despite the place that entropy assumes within modern thermodynamics. Irreversible thermodynamics, for example, is founded upon the principle of entropy production, but what exactly is being produced and how is not clear. Yet, if we are to assert that entropy is a physical property of a system in a given physical state and therefore increases when the system changes from one state to another, we are entitled to ask: What is the mechanism? What is the cause and what is the effect? If we cannot answer those questions, do we conclude that entropy is not a physical property of a physical system, despite the acceptance of such ever since Clausius?

The idea that entropy is a property of matter is one of the founding principles of irreversible thermodynamics [5]. An entropy per unit mass is defined and the total entropy of the system can change through the transport of matter by diffusion and convection. More recent developments in irreversible thermodynamics have attempted to treat reversible and irreversible processes alike by mapping a space of non-equilibrium states on to the space of equilibrium states and by defining non-equilibrium quantities such as a contact temperature, which determines the entropy exchange with the environment arising from heat flow [6].

These are complex mathematical ideas that have the potential to treat complex irreversible phenomena, but still they depend on the notion that entropy is a property of matter and that it is produced in an irreversible process. In addition, a question arises over the physical nature of a non-equilibrium state. By the very definition, a non-equilibrium state is transient. Properties such as pressure and temperature are equilibrium concepts so, whilst it might be possible to define local effective quantities that allow for calculation of heat flow or work performed during a particular process, it is not possible generally to define effective values for the state itself. Quite what a non-equilibrium state is and how it maps on to equilibrium states are questions that would appear to be answerable with reference to mathematics rather than physics.

So, what is entropy? Leff recently explored the energy spreading metaphor as a way of understanding entropy [7], and was unable to come to a firm conclusion. Leff’s paper is interesting in as much as it has primarily a pedagogical focus and his inability to come up with a clear conceptual model of entropy to help students understand the concept is telling. He described the energy spreading metaphor as an interpretive tool but raised many questions which remained unanswered. More significantly, Leff quoted two authors, Čápek and Sheehan, from their book *Challenges to the Second Law of Thermodynamics—Theory and Experiment* [8]: “Entropy remains enigmatic. The more closely one studies it, the less clear it becomes. Like a pointillism painting whose meaning dissolves into a collection of meaningless points when observed too closely, so too entropy begins to lose meaning when one contemplates it at a microscopic level”. The idea that, microscopically, entropy loses its meaning is profoundly disturbing, but in this author’s view, the enigmatic nature of entropy goes beyond a loss of meaning at the microscopic level to a multiplicity of meanings at the macroscopic level. It seems impossible to settle on a single interpretation that satisfies all circumstances.

Consider, for example, the notion of additivity. One of the axioms of the non-equilibrium approach in [6] is that the entropy of the subsystem and its reservoir are additive. The author has investigated the connection between thermodynamic entropy and accessible states in simple systems and shown that in an ideal gas it is possible to define a single-particle entropy in a system thermally connected to a heat reservoir [9]. The *N*-particle entropy is just the sum of single particle entropies. Entropy is therefore additive, but it derives from the possible states of motion of the particle rather than some intrinsic property of matter.

Additivity also features in the Gibbs paradox. This highlights a problem that stubbornly refuses to be solved even now, well over 100 years after Gibbs published his seminal work on statistical mechanics. Two gases at the same temperature and pressure, say *A* and *B*, each with *N* particles, are contained in the different halves of a chamber separated by a partition. On removal of the partition the gases inter-diffuse. Both the thermodynamic and statistical entropies increase. However, if the two halves contain the same gas, whether *A* or *B*, the statistical entropy increases, but the extensivity property of entropy requires the total thermodynamic entropy to be the sum of the entropies of the two halves.

The increase in entropy for different gases can also be arrived at by considering the expansion of each gas into an equal empty volume upon removal of the partition, which implies that the entropies of the two different gases are additive. For identical gases, the same final state could be achieved by having all 2*N* particles in one half of the system and allowing the gas to expand freely. As the free expansion is an irreversible process, the entropy must increase, which means that the entropy of 2*N* particles in a given volume at a given temperature must be less than twice the entropy of *N* particles in the same volume at the same temperature. Therefore, the thermodynamic entropy of a single gas within a given volume is not additive.

It is possible that the Gibbs paradox is not a paradox at all. A true paradox implies an inconsistency or contradiction, but the Gibbs paradox simply points out that the statistical and thermodynamic entropies calculated under a given set of circumstances are sometimes different and sometimes the same, depending on the distribution used. A statistical entropy can be defined for any proper probability distribution regardless of whether the distribution has any connection with thermal phenomena. Certain distributions, such as the Maxwellian velocity distribution, yield entropy functions that appear to have identical mathematical forms to thermodynamic entropy [9] but others do not. The Gamma distribution gives the probability that an ideal gas in thermal contact with a reservoir is in a given energy state [10], but its entropy looks nothing like thermodynamic entropy. The Boltzmann entropy does not appear to be equivalent to the thermodynamic entropy under all circumstances [11], so the fact that mathematical forms appear to be identical under some circumstances is not in itself proof that the two are equivalent.

The free expansion of a gas is also problematic. Clausius considered this process in his Sixth Memoir when he developed the function which he later called entropy. He recognised that there is a state function and as such it must increase during a free expansion. The question which Clausius did not consider, and which, to this author’s knowledge has not been considered since, is whether a state function, which is a mathematical consequence of differential geometry, is also a property of a body in that same thermodynamic state. There are two essential difficulties. First, the failure to attach a physical meaning to thermodynamic entropy as a property of a body means that we have no idea what is increasing, what the physical mechanism is, or what the physical consequence is. Secondly, there is no change in energy during the free expansion. It would not be fair to say that Clausius overlooked conservation of energy in his thinking on entropy because the concept of energy was not well developed at the time, but his thinking was not consistent with the notion of energy conservation as it later came to be understood.

The modern reader attempting to understand Clausius’s writing faces two difficulties: first, his writing style is cumbersome and consequently his meaning is not always clear and, second, his view of heat does not correspond to the modern view. The idea of “heat as motion” was taken literally by Clausius and he wrote of heat as though it was what was later understood as internal energy. Although Clausius was aware of the notion of internal energy, and indeed he appears to have been the first to write the mathematical form of the first law [1] (p. 29), he seems to have regarded heat as the more fundamental quantity. In the First Memoir, for example, he described the heat absorbed by a gas as being decomposed into two portions, one of which is *U* and the other external work. *U* comprised, “the sensible heat and the heat necessary for internal work”. In the Fourth Memoir [12] (p. 115), he described a quantity of heat absorbed by a body, *Q*, as being divided into three parts: the first is, “employed in increasing the heat actually existing in the body”, the second producing the “interior” work and the third producing the “exterior” work. Interior work may be understood as the work performed by particles moving against inter-particle forces and external work is accomplished by a gas acting on a piston or some other device.

The idea of internal work was central to his development of entropy. As described in the Ninth Memoir [13] (p. 355), entropy is given by

(1) $$\int \frac{dQ}{T}=\int \frac{dH}{T}+\int dZ$$

Clausius described *H* as denoting, “the heat actually present in the body”. This, he claimed, depended, “solely on the temperature of the body and not on the arrangement of its parts”, and the first integral on the right therefore leads to, “a magnitude which is perfectly defined by the present condition of the body”. The quantity, *Z*, was defined in the Sixth Memoir as the disgregation and, “depends on the arrangement of the particles of the body”. It was invoked by Clausius to account for internal work against what he called, “the separative force of heat”. These three quantities, *H*, *T* and *Z*, either defined, or were defined by, the thermodynamic state and Clausius therefore regarded entropy as a property of a body.

Neither *Z* nor *H* are recognised in modern thermodynamics. *H* is effectively the kinetic energy of the particles and the idea of dividing heat out into separate components was criticised by Maxwell 1876 [14]: “With respect to our knowledge of the condition of energy within a body, both Rankine and Clausius pretend to know something about it. We certainly know how much goes in and comes out and we know whether at entrance or exit it is in the form of heat or work, but what disguise it assumes when in the privacy of bodies, … is known only to R., C. [Rankine and Clausius] and Co”.

The quantity, *Z*, has disappeared from the thermodynamic lexicon but is central to the difficulty over energy. Clausius saw no essential difference between cyclic and non-cyclic processes: “since there is no essential difference between interior and exterior work, we may assume almost with certainty that a theorem which is so generally applicable to exterior work cannot be restricted to this alone, but that, where exterior work is combined with interior work, it must be capable of application to the latter also” [15] (p. 219). The theorem applicable to exterior work was the idea that, around a closed cycle

(2) $$\oint \frac{dQ}{T}\leq 0$$

The inequality applies to irreversible cyclic processes and, in the Sixth Memoir, Clausius sought an inequality equivalent to (2) that would apply to irreversible, non-cyclic processes based on the combination of interior and exterior work. Having previously incorporated internal work into *U*, he therefore split *U* back into its two constituent components: the actual heat present in a body, *H*, and the internal work, *I*. He then combined the internal and external work into the disgregation, *Z*, and noted that in an irreversible process the external work is always algebraically less than the reversible work. Therefore

(3) TdZ=dI+PdV≥dI+dW

The entropy change therefore becomes

(4) $$dS=\frac{dQ}{T}=\frac{dH}{T}+dZ\geq \frac{dU}{T}-\frac{dW}{T}$$

**Figure 1:**
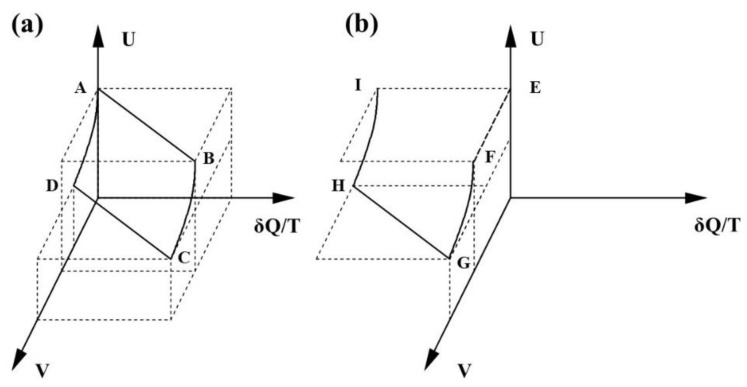
A 3-D representation of changes in δQ/T according to Equation (2) for (**a**) the Carnot cycle and (**b**) the same cycle with the initial isothermal expansion (AB) replaced by a free expansion (EF).

In order to illustrate these ideas, Clausius discussed in the Sixth Memoir replacing the isothermal expansion in a Carnot cycle with the free expansion. *TdZ* is equivalent to *PdV as dQ = dW =* 0 and the entropy in Equation (4) becomes a state function. However, the cycle is also irreversible and, according to Equation (2), the sum of all changes in *dQ/T* must be negative. This is illustrated in Figure 1, which shows a schematic 3-D plot of *U*, *V* and *δQ/T* for both the reversible and irreversible cycles. The Carnot cycle is shown in (a) by the path *ABCD* and the cycle incorporating the free expansion is shown in (b), by the path *EFGHI*. It should be apparent that the *dQ/T* in Equation (2) must be different from the *dQ/T* in Equation (4). The latter is a state function and the former self-evidently is not.

This is a distinction that Clausius did not recognise. Equation (2) applies to cyclic process and the application is strict. *dQ/T* is negative because a nett outflow of heat is required to restore the initial condition. Therefore, a negative value of *δQ/T* in Equation (2) is fully consistent with energy conservation whilst *dQ/T* in Equation (4) is not. *TdS* has the units of energy and increases by *PdV* during the free expansion, but there would appear to be no physical mechanism, no cause and no measurable effect.

This difficulty persists to the present day. We have

(5) $$TdS=dQ_{rev}=dU-dW_{rev}$$

However, *dU* is also defined by the energy changes that actually occur

(6) dU=dQ+dW

Therefore

(7) $$TdS=dQ-\left(dW_{rev}-dW\right)\geq dQ$$

For irreversible processes, the magnitude of *dW_rev_* exceeds *dW* and the sign of work processes relative to change in volume means that the difference will always be positive, hence *TdS* > *dQ*.

Clausius justified the inequality in Equation (4) in the Sixth Memoir [15] (p. 223) by claiming: “The law does not speak of the work which the heat **does**, but of the work which it **can do**; and similarly, in the first form of the law, it is not of the resistances which heat overcomes, but of those which it **can overcome** that mention is made.” Clausius himself emphasised the terms in bold. The resistance that heat can overcome is an external pressure, *P,* that matches the internal pressure, resulting in work *PdV*. In Clausius’s view, the effect of such work was to reverse the separation between particles and *PdV* was therefore a measure of the “separative force of heat”. However, the first law is not concerned with potentialities, but with work that *is* performed and heat that *does* flow. *PdV* is a work term and therefore implies a change in energy, but it does not correspond to work performed, and therefore the changes in energy, in an irreversible process.

The preceding has highlighted some of the features of entropy that make it such an enigmatic quantity and traced them back to the origins of the concept and the deep connection between entropy and reversibility in Clausius’s thinking. In particular, Equation (5) shows explicitly how entropy changes depend on the notion of reversible work. Clausius’s separation of the internal energy into “actual heat” and internal work has effectively been reversed, leaving reversible work as a separate component of the entropy.

Clausius’s concept of reversible work was based on reversing the separation between particles. Although he recognised that when the internal and external pressures are equal no work can be performed, he also argued that the separation of particles could be so closely reversed that it is possible to imagine PdV as a limiting case. However, if a step change in external pressure is required for work to be performed, it is possible that work is intrinsically irreversible [16]. The author has shown by means of kinetic simulations [17,18] that internal mechanisms of damping exist in an ideal gas, and these suggest that microscopically reversible processes do not exist. This has implications for the Carnot cycle.

Modern thermodynamics reflects Clausius’s view that a reversible cycle is made up of processes which are themselves reversible and, on the face of it, it is difficult to see how a reversible cycle is possible if work processes are intrinsically irreversible. In this paper, an alternative conception of the Carnot cycle that does not depend on such microscopically reversible processes is presented. The internal damping mechanisms identified in [17,18] are incorporated into rate equations that couple the rate of exchange of energy with the environment, either through work or heat flow, to the rate of internal damping. These are solved numerically. Intrinsic irreversibility is confirmed, but under idealised conditions of no damping, the system maps out a trajectory through the space of equilibrium states, which is reversed as the piston motion is also reversed. With appropriate adjustments to the external conditions, an ideal gas can be made to execute a reversible Carnot cycle. The cycle is dynamic rather than quasi-static in as much as the piston is always in motion and at no time settles in a static equilibrium state. The connection between entropy changes in such a cycle and the mathematical notion of entropy as a state function are discussed.

## 2. The Thermodynamics of an Ideal Gas Based on Rate Equations

**Figure 2:**
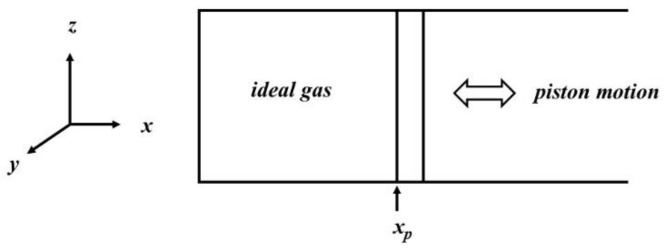
A schematic of the thermodynamic system modelled in this paper.

Consider the situation depicted in Figure 2. A cylinder containing an ideal gas is aligned with the *x*-axis of a 3-D Cartesian coordinate system and open at one end. It has a piston with its gas-facing surface located instantaneously at position *x_p_*. The piston is moving with a velocity *v_p_*, which can be either positive or negative. Taking the gas in the left-hand side of the piston only, at any instant the kinetic energy of a single particle can be regarded as the algebraic sum of three terms associated with the component of the motion along each axis. The sum over all particles of each of the three separate terms is just the total kinetic energy. As the gas is ideal, there is no interaction with other particles or with the walls except through collisions and within the piston the internal energy is equal to the kinetic energy: that is

(8) $$U=\overset{n}{\underset{j=1}{\sum }}\frac{1}{2}mv_{j}^{2}=\overset{n}{\underset{j=1}{\sum }}\frac{1}{2}m\left(v_{jx}^{2}+v_{jy}^{2}+v_{jz}^{2}\right)=E_{x}+E_{y}+E_{z}$$

Equilibrium is defined by

(9) $$E_{x}=E_{y}=E_{z}=\frac{U}{3}$$

Apply this now to the rate at which work is performed. In reality, the interaction between the particles of a gas and the wall of a container, including the surface of a piston, are quite complex. In kinetic theory it is usual to regard collisions between the particles and the wall as elastic, but a moment’s reflection will show that this cannot be the case. Whatever might be happening at the microscopic scale, whether particles are first adsorbed and then emitted at random or interact directly with vibrational modes (phonons) in the chamber wall, macroscopically it must be possible for energy to be transferred from the wall to the gas and vice versa, depending on the relative temperatures. Otherwise, it would not be possible for a gas at a different temperature from the walls of a chamber to thermalise with the chamber. For a gas interacting with a moveable piston there are two effects to consider: an exchange of heat between the gas and the piston and the transfer of momentum in order to allow the piston to move. As the total energy must be conserved, the two are connected and for the purposes of modelling the piston motion it is simpler to assume that no heat is transferred. That is to say, the collisions are elastic and only the conservation of momentum need be considered.

It follows from the preceding that only the component of particle velocity in the direction of the motion of the piston is affected and the component of velocity perpendicular to the plane of the piston remains unaffected. It will be apparent, therefore, that the action of gas particles on the piston will lead to a loss of equilibrium within the gas in as much as

(10) $$E_{x}\neq \frac{U}{3}$$

Two consequences follow from this. First, there must be some mechanism of relaxation within the gas to restore equipartition and second, while the gas is out of equilibrium the pressure acting on the piston will be different from the equilibrium pressure given by the equation of state. The effective pressure can be found by looking at the mechanics of a collision between a gas particle, say an atom, with mass *m_a_* and velocity *u_a_*, and a piston, with mass *m_p_* and velocity *u_p_*. Conservation of both momentum and kinetic energy leads to well-known expressions for the velocities after the collision, respectively *v_a_* and *v_p_*, given by

(11a) $$v_{a}=\left(\frac{m_{a}-m_{p}}{m_{a}+m_{p}}\right)u_{a}+\frac{2m_{p}}{m_{a}+m_{p}}u_{p}$$

And

(11b) $$v_{p}=\left(\frac{m_{p}-m_{a}}{m_{a}+m_{p}}\right)u_{p}+\frac{2m_{a}}{m_{a}+m_{p}}u_{a}$$

The mass of the piston is of course much greater than the mass of the particle so for the atom

(12) $$v_{a}=2u_{p}-u_{a}$$

If *u_p_* is positive, i.e., moving in the same direction as the impinging atom, then $\left|v_{a}\right|<\left|u_{a}\right|$. In other words, a gas particle loses energy on each collision. Generally, *u_a_ >> u_p_*, so the effect is quite small for each individual particle, but as even a small volume contains a very large number of particles, the collective effect is to cool the gas as it works on the piston. On the other hand, if *u_p_* is negative and the piston is moving towards the gas particles, the gas gains energy as work acts on it.

By Newton’s third law, the magnitude of the force acting on the piston is just the rate of change of momentum of the atoms. The change in momentum of a single atom is

(13) $$m_{a}v_{a}-m_{a}u_{a}=2m_{a}u_{p}-2m_{a}u_{a}$$

Suppose *N*′ atoms collide with the piston in a time Δ*t*. The direction of the force is in the opposite direction to the change in momentum of the gas particles, so

(14) $$F=-\frac{N\prime }{\Delta t}\left(mv_{a}-mu_{a}\right)=\frac{N\prime }{\Delta t}2m_{a}\left(u_{a}-u_{p}\right)$$

In order to find *N′* assume the gas contains 6*N* atoms/unit volume, all travelling in different directions with a range of speeds. A sample of those 6*N* atoms close to the piston will collide with it. If the probability of finding an atom with a component of velocity in the positive x-direction between *u* and *u*+*du* is *p*(*u*)*du*, then it is clear from (14) that the total force taken over all the atoms in *N*′ will depend on the average speed provided we ignore those atoms for which *u_a_* < *u_p_*, as these do not collide with the piston and therefore do not contribute to the force. Strictly, excluding these atoms would require some renormalisation of the probability distribution, but for simplicity we ignore that and move to a model in which we regard all the atoms as travelling with the same average speed. We can therefore divide the 6*N* atoms into *N* atoms each travelling in the positive and negative *x*, *y* and *z* directions. The average speeds in each of the different directions will in general be different and in both the positive and negative *x* directions there could also be a spatial dependence as atoms that have collided with the piston propagate through the system. Collisions between particles will smooth out these differences as energy is redistributed among the different degrees of freedom, but any differences in the total momentum in the atoms induced by collisions must remain.

If all the atoms within a short distance ∆*l*, travelling with an average speed *u_a_*, collide with the piston in time ∆*t*, then clearly $\Delta t=\frac{\Delta l}{u_{a}}$ and $N\prime =NAu_{a}\Delta t$, where *A* is the area of the piston.

Therefore

(15) $$P=\frac{F}{A}=2Nm_{a}u_{a}^{2}\left(1-\frac{u_{p}}{u_{a}}\right)$$

Putting

(16) $$N\left(\frac{1}{2}m_{a}u_{a}^{2}\right)=E_{x}^{+}$$

where *E_x_^+^* is the kinetic energy of the atoms associated with motion in the positive *x* direction, i.e., towards the piston, we have

(17) $$P=4E_{x}^{+}\left(1-\frac{u_{p}}{u_{a}}\right)$$

Particles that have impacted on the piston will have had their momentum altered, as described above, and, strictly, *E_x_^+^* will be different from the kinetic energy of the atoms moving in the opposite direction, denoted by *E_x_^−^*. In a kinetic model, this would automatically be accounted for through knowledge of the individual velocities of the particles as they propagate through space and collide with other particles and the walls of the chamber. In a macroscopic formulation, the loss of information about individual momenta and their changes in both space and time means that it will be very difficult, perhaps impossible, to keep track of the differences between *E_x_^+^* and *E_x_^−^* in both time and space. Out of necessity, the differences between *E_x_^+^* and *E_x_^−^* are ignored and instead *E_x_*, the total energy associated with the velocity components in both the positive and negative directions, is used. In effect,

(18) $$E_{x}=2E_{x}^{+}$$

And

(19) $$P=2E_{x}\left(1-\frac{u_{p}}{u_{a}}\right)$$

Therefore, the effective pressure acting on the piston is not the intensive quantity in classical thermodynamics but a one-dimensional, dynamic quantity that depends not only on the properties of the particles via their energy, but also on the speed of the piston.

If the external pressure on the right-hand side of the piston in Figure 2 is *P_ext_*, the nett force on the piston is

(20) $$F=\left(P-P_{ext}\right)A$$

The rate at which work is performed on the piston is

(21) $$\frac{dW_{p}}{dt}=\left(P-P_{ext}\right)A\frac{dx_{p}}{dt}$$

The two terms on the right-hand side of Equation (21) are recognisable as the rate of work performed on or by the gas, depending on the sign of *dx_p_*, PdVdt, and on or by the external load, PextdVdt.

For a gas in equilibrium, the energy is partitioned equally among the degrees of freedom, but changes in *E_x_* at the piston will change the internal energy and the system will be out of equilibrium, in the sense given by Equation (10). The exchange of energy between the different degrees of freedom, say *E_x_*, and, for example, *E_z_*, can be described by the rate equation

(22) $$\frac{dE_{xz}}{dt}=\propto_{z}\left(\frac{U}{3}-E_{z}\right)$$

An identical expression applies to scattering into *E_y_* denoted by the process variable, *E_xy_*. The constant, *α_z_*_,_ in (22) is a variable parameter equivalent to an inverse time constant. Thus, for example, if $E_{z}<\frac{U}{3}$ energy is scattered out of the *x*-direction and into the *z*-direction. Energy conservation requires

(23) $${\left(\frac{dE_{x}}{dt}\right)}_{scattering}=-\left(\frac{dE_{xz}}{dt}+\frac{dE_{xy}}{dt}\right)$$

The subscript on the left-hand side denotes that only changes to *E_x_* brought about by scattering are being considered in (23). In addition to scattering, heat can flow in from the external walls and affect the total energy. Consider heat flow from the *y-z* plane into *x*, i.e., the wall opposite the piston on the left of Figure 2. This affects *E_x_*. The one-dimensional rate of heat flow can be defined as

(24) $$\frac{dQ_{x}}{dt}=\propto_{h}\frac{Nk_{B}}{2}\left(T_{wall}-T_{x}\right)$$

where *T_wall_* is the wall temperature and is equal to the starting temperature of the gas, $\propto_{h}$ is again a variable parameter equivalent to an inverse time constant, $\frac{Nk_{B}}{2}$ is a one-dimensional heat capacity and the 1-D equivalent temperature, *T_x_*, can be defined from kinetic theory as

(25) $$T_{x}=\frac{2E_{x}}{Nk_{B}}$$

*T_x_* is the effective temperature associated with motion in the *x*-direction and is greater than, or less than, the equilibrium temperature of the gas according to whether *E_x_* is greater than, or less than, $\frac{U}{3}$. Heat flow from walls in the *y-x* and *z-x* planes into *E_z_* and *E_y_*, respectively, can be expressed in the same manner. Under these assumptions, namely that:

1. Heat flows into the system from walls through inelastic collisions;
2. The collisions with the piston are perfectly elastic and therefore only the x component of velocity is affected;

Then

(26a) $$\frac{dE_{x}}{dt}=\frac{dQ_{x}}{dt}-\frac{dW_{p}}{dt}-\frac{dE_{xy}}{dt}-\frac{dE_{xz}}{dt}$$

(26b) $$\frac{dE_{y}}{dt}=\frac{dQ_{y}}{dt}+\frac{dE_{xy}}{dt}$$

(26c) $$\frac{dE_{z}}{dt}=\frac{dQ_{z}}{dt}+\frac{dE_{xz}}{dt}$$

These equations can be solved numerically by a simple forward difference time step, with the time step, *δt*, being reduced until a stable solution is achieved. Then, the heat entering the system from the *y-z* wall is

(27) $$\delta Q_{x}=\propto_{h}\delta t\frac{Nk_{B}}{2}\left(T_{wall}-T_{x}\right)$$

It is evident that the upper limit corresponds to ideality: that is, for $\propto_{h}\delta t=1$, heat flow is instantaneous and the gas temperature remains constant and, for $\propto_{z}\delta t=1$, equipartition occurs instantly and the gas is always in internal equilibrium. In reality, both *α_h_* and *α_z_* are properties of the gas and its interaction with its surroundings and are therefore independent of *δt*, but, in as much as they are unknown, they can be varied at will in the simulations. This does mean, however, that this approach is not suited to an exploration of the dynamical evolution of such systems as the evolution depends not only on the choice of scattering parameters, *α_h_* and *α_z_*, but on knowledge of the spatial variation of both energy and particle densities, as well as the flow of momentum. The more complex computational approaches of irreversible thermodynamics [19] are probably better suited to this kind of in-depth exploration. Here, the emphasis is on the final state and, as will be shown in the next section, the final state depends only on the equality of internal and external pressures and is independent of the dynamics of the system.

## 3. Numerical Solution of the Thermodynamic Rate Equations

### 3.1. Step Changes in Pressure

We start with a small but finite change in external pressure in order to show, first, that the macroscopic formulation presented above reproduces the results of the kinetic simulation in which it is based, and, second, to investigate the nature of irreversibility in a simple thermodynamic system. Equations (19)–(27) were solved numerically using purpose-written code run in a 64-bit implementation of Python 3.8 for a system comprising an ideal gas with an atomic mass of 20, the same as Ne, initially at standard temperature and pressure. The choice of volume and piston starting position were arbitrarily chosen to correspond to a cylinder of cross-sectional area 1 cm^2^ with a piston initially located at *x_p_* = 10 cm. The mass of the piston was calculated by specifying its thickness as a variable and using the density of aluminium as a typical metal from which such a piston might be made.

The mass of the atoms allows the calculation of a mean particle speed in Equation (19). As *N*/3 particles are assumed to be travelling along the *x*-axis, this mean speed was updated with each change in *E_x_* according to

(28) $$v_{x}={\left(\frac{6E_{x}}{Nm_{a}}\right)}^{\frac{1}{2}}$$

The rate equations were solved by a simple process of a forward time step so that the differentials become finite differences in the manner of Equation (27). Equation (20) was used in conjunction with Newton’s second law to calculate the acceleration of the piston. With a small enough time step, the acceleration can be assumed constant and the distance travelled by the piston in the time step is easily computed. The work performed on or by the gas was then calculated from knowledge of the pressure and the change in volume, and *E_x_* adjusted accordingly. For simplicity, only heat flow from the rear wall in Figure 2 was permitted and the only changes to *E_y_* and *E_z_* considered were those due to scattering, with the additional assumption that *E_z_* = *E_y_* at all times. For adiabatic processes, *α_h_* was set to zero.

This assumption makes clear the idealized nature of adiabatic processes. Ordinarily, the walls of any container will have a much larger heat capacity than the gas contained within it and can act as a reservoir, exchanging energy with a gas within, even if it is lagged to prevent heat flow from outside. The assumption of *α_h_* = 0 is an idealization that limits the system under consideration to the gas and the piston alone. The only interaction between the piston and gas is through the exchange of momentum, which changes the temperature of the gas but not the piston. The piston is therefore a mechanical device which acts as a repository for the difference in work performed within the gas and the exterior pressure. Setting *α_h_* > 0 is a device that connects the gas to a heat reservoir and allows the final temperature to equal the initial temperature.

**Figure 3:**
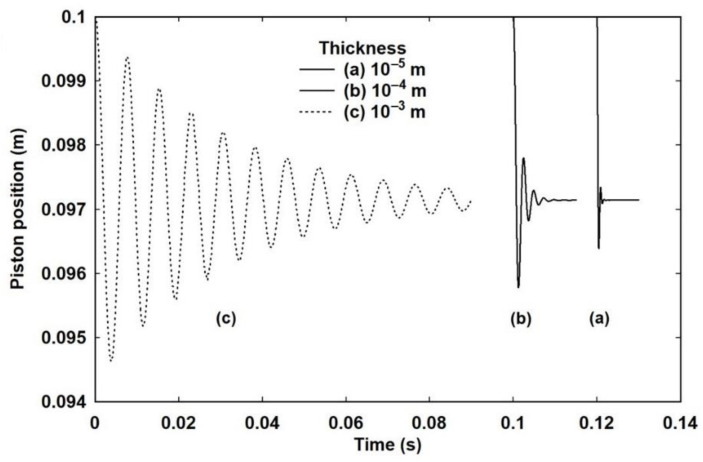
The effect of piston mass on the decay time in an adiabatic process. For computational purposes, the mass is determined by the thickness using the density of aluminium (2700 kg m^−3^) as a typical metal from which such a piston might be made. The origins of curves (a) and (b) are offset for clarity but the timescale is unchanged.

The initial temperature was set to 295 K and the pressure to 101,325.0 Pa. This allows the total number of particles to be calculated and the equilibrium starting value of *E_x_*. Both expansion and compression from this starting state were modelled by setting the external pressure to be a factor (1 ± *β*) of the internal pressure, where −0.1 ≤ *β* ≤ 0.1 in steps of 0.01. The time step was set to *δt* = 10^−9^ s, which is small enough to give stable results. Figure 3 shows the piston position as a function of time for *β* = 0.5, corresponding to an external pressure of 106,391.25 Pa, with *α_h_* = 0 and *α_z_* = 10^4^, for three different piston thicknesses of 10^−5^, 10^−4^ and 10^−3^ m. For clarity, the data for the thinner pistons has been offset to 0.12 and 0.10 s, respectively, but the timescales remain the same. It is apparent that the heavier the piston, the longer the decay. It is not just that the piston moves slower, hence the larger period of the motion, but also that there are many more oscillations within the decay. For the thinnest piston, 10^−5^ m, corresponding to a mass of 2.7 mg, there is essentially only one oscillation and the movement from the starting position to the minimum looks almost like a vertical line on this time scale. All three cases settle at the same final position and, for computational convenience, and unless mentioned otherwise, the piston thickness was set at 10^−4^ m for all other calculations. It is easily verified that the change in internal energy at the final position of 0.097143 m is identical to the external work performed.

**Figure 4:**
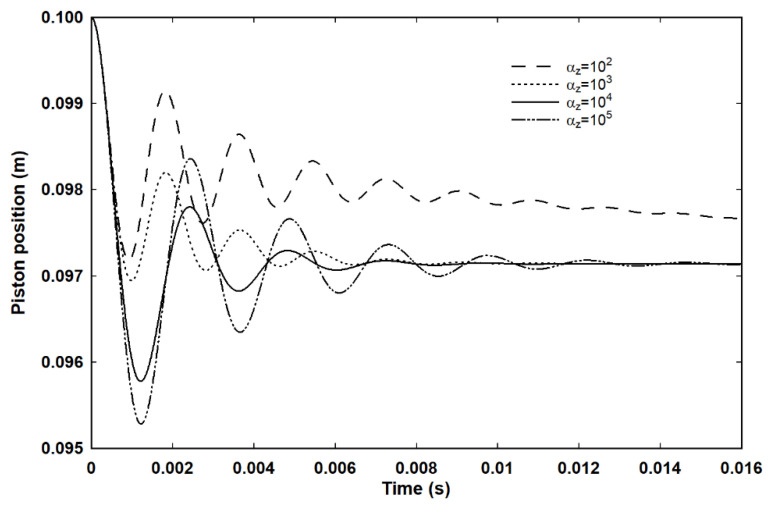
The effect of the energy scattering parameter, α_z_, on the decay of the piston motion for an adiabatic process with an external pressure of 1.05 × atmospheric. The solid line, α_z_ = 10^4^, was used for the majority of simulations in conjunction with a time step of 10^−9^ s.

**Figure 5:**
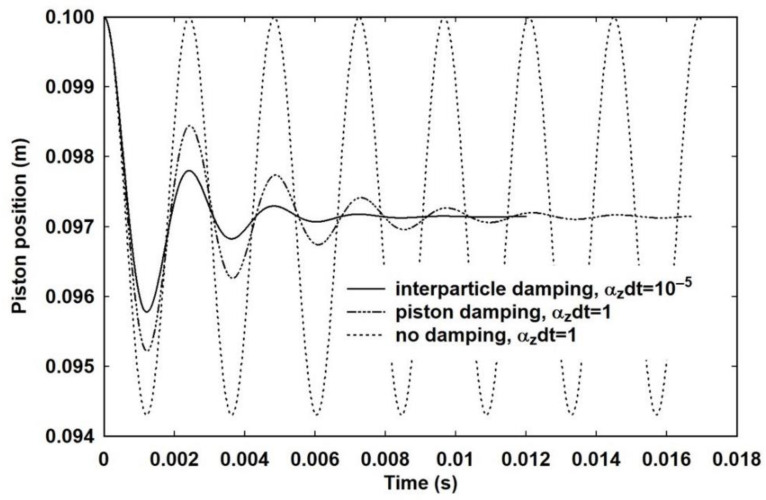
A demonstration of piston damping for an adiabatic process with an external pressure of 1.05 × atmospheric. The solid line is the same decay as in Figure 3 for comparison. Elimination of the dependence of the effective pressure on the speed of the piston gives undamped oscillations.

Figure 4 shows the piston motion for four values of *α_z_*, with *α_z_* = 10^2^ having the slowest decay of the oscillation followed by a steady decay to the equilibrium position. The data in Figure 4 has been truncated in time, but the downward trend for the piston position for *α_z_* = 10^2^ is evident. The explanation for this is provided by Figure 5, which shows that, for *α_z_δt* = 1, corresponding to instantaneous equipartition of energy such that the gas can be considered be in internal equilibrium, the motion is still damped. The cause can be traced to the dependence of the pressure on the speed of the piston in Equation (19). By contrast, omission of this term with *α_z_δt* = 1, so that the effective pressure is just 2*E_x_* and equivalent to the equilibrium pressure given by the equation of state, leads to undamped motion. Indeed, a simulation based on the internal energy and the corresponding equilibrium pressure yields the same indefinite oscillation. For *α_z_* = 10^2^ then, the piston motion has been damped before full equipartition occurs. The gas is therefore out of equilibrium, but scattering out of *E_x_* into *E_y_* and *E_z_* continues according to Equations (26a)–(26c) and the system very slowly relaxes to equilibrium. As it does so, the effective pressure acting against the piston also reduces and, if left long enough, the system settles at the expected position.

Here, this effect is called piston damping because it depends on the piston motion rather than energy relaxation by conduction or scattering. Confirmation of this explanation is provided in Figure 5, which shows piston damping for three different conditions:

1. Both *α_z_* and *α_h_* set to zero, corresponding to an adiabatic process with no scattering out of *E_x_* into *E_y_* and *E_z_*;
2. Both *α_z_* and *α_h_* set such that both *α_z_δt* = 1and *α_h_δt* = 1, corresponding to ideal isothermal;
3. *α_z_* is set such that *α_z_δt* = 1 but *α_h_* = 0, corresponding to ideal adiabatic, which is the same situation as shown in Figure 4.

In all three cases the piston should oscillate indefinitely. For conditions 2 and 3 above, corresponding to ideal isothermal and adiabatic, respectively, the gas is effectively in equilibrium and piston damping causes the piston to settle in the expected position. For case 1 above, the lack of any scattering mechanism, including equipartition, means that *E_x_* is always different from both *E_y_* and *E_z_* and the system settles in an artificial state.

**Figure 6:**
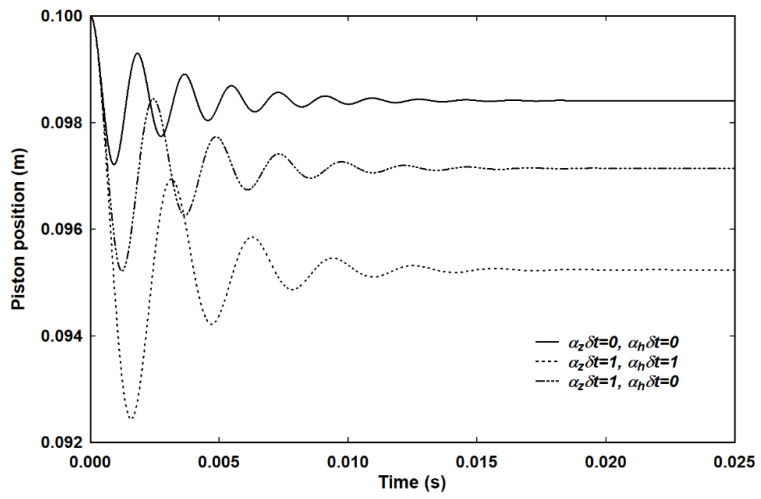
Piston damping for an external pressure of 1.05 × atmospheric and other damping mechanisms eliminated. The curves correspond to an adiabatic process with no equipartition, an adiabatic process with instantaneous equipartition and an isothermal process with instant heat flow and instant equipartition.

Piston damping is therefore seen to be a real computational effect that arises from the velocity-dependence of the pressure in Equation (19). Whether it corresponds to a real, physical effect is as yet unclear. By contrast, inter-particle collisions and heat flow, represented by *α_z_* and *α_h,_* respectively, represent processes that have been shown to give rise to damping in kinetic simulations [17,18]. When inter-particle collisions were switched off, undamped oscillations were observed, but in the macroscopic model, as shown in Figure 6, switching off the inter-particle collisions still gives rise to damped motion. In a kinetic simulation, the velocity dependence of the effective pressure should automatically be accounted for through the speed of the particles, so the lack of any damping observed at the microscopic level is contrary to the observation of damping in the macroscopic formulation. It is entirely possible, therefore, that the effect is a consequence of the loss of microscopic information and the implicit averaging that necessarily occurs in the transformation from a microscopic to a macroscopic view. It is for this reason that the simulations reported in this paper rely on one or both of *α_z_* and *α_h_* as sources of damping.

**Figure 7:**
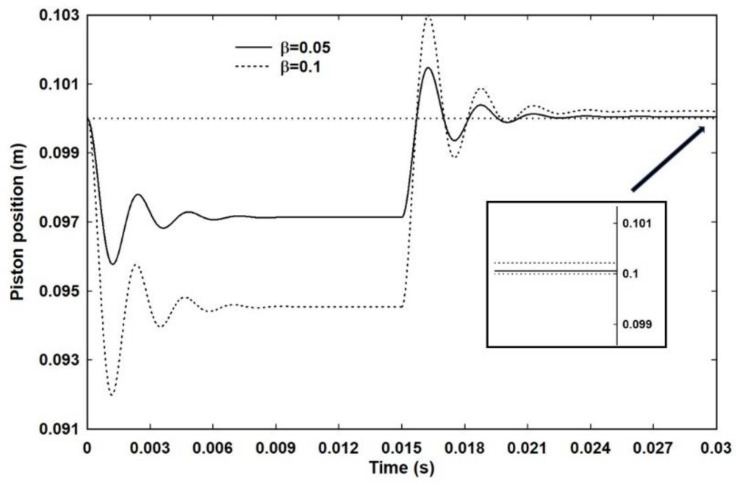
Two 2-step processes corresponding to a step increase in external pressure followed by a return to the starting external pressure. The starting position of the piston is indicated by the dotted line at a position of 0.1 m. The inset shows clearly that the system does not return to the starting state but, as would be expected, the final volume is greater than the initial volume.

**Figure 8:**
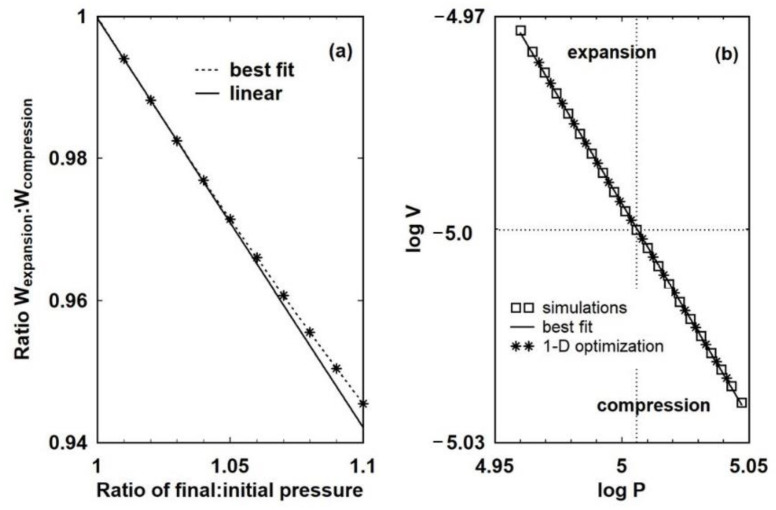
The ratio of work performed in expansion to the work performed in compression for a two step process in which compression is followed by expansion (**a**). For small changes, the ratio of works is approximately linear with the ratio of initial to final pressure in the compression stage and always less than unity. In (**b**) a plot of log V against log P yields a straight line with a best fit slope of −0.6002, which is effectively the inverse of γ, the ratio of specific heats.

It was argued in the introduction that the existence of internal damping mechanisms means that work processes are intrinsically irreversible: that is, a change of state brought about by a finite change in the external pressure will not be reversed if the external pressure is reset to the original condition. This is illustrated in Figure 7, which shows two such two-step processes for β = 0.05 and 0.1 with *α_z_* = 10^4^. As expected, the system expands to a larger volume upon reversal of the external pressure. The inset shows this more clearly. Although the difference is small, it is only because the initial change is small, but it is nonetheless real and within the limits of computational accuracy, even for β = 0.05. There is, of course, a limit as to how small a change can be modelled on a computer with a set numerical resolution, so rather than attempt to model very small changes, instead the data from slightly larger changes is extrapolated back. Thus, Figure 8a shows the ratio of work performed in expansion to the work performed in compression plotted against 1 + β. The data appears linear over a small range of β, but is in fact best described by a gentle curve. There is no sense of asymptotic behaviour or the ratio becoming unity as β moves towards zero, such that the work performed in either direction approaches equality and the process becomes effectively reversible. The ratio only approaches unity for β = 0, in which case there is no change in pressure and therefore no change of state. This is exactly what would be expected for even very, very small changes in a system that is intrinsically irreversible. There are no circumstances in which a finite change of external pressure leads to a return to exactly the same state on restoration of the external pressure.

This is further supported by Figure 8b, which shows a plot of *log V* against *log P* for the final state for −0.1 ≤ β ≤ 0.1 in steps of 0.01, a piston thickness of 10^−4^ m and *α_z_* = 10^4^. The axes are plotted this way because in the simulation the pressure is the independent variable. Also shown is the line of best fit that has a slope of −0.6009, which is effectively the inverse of γ, the ratio of specific heats. However, this is an average. Closer examination of the data reveals that there are two slightly different slopes, one corresponding to compression (−0.6149) and one corresponding to expansion (−0.58960). In [16], a mathematical argument was made for intrinsic irreversibility using a binomial expansion of $PV^{\gamma }=k$ for a small step change in *P* and a corresponding step change in *V*. This argument is open to the criticism that this expression is derived using the notion of reversibility, and whilst the existence of two slopes shows that the relationship linking states in the process of compression is different from that in expansion, on average the relationship holds for irreversible processes over the limited range of expansion and compression for which the binomial expansion would apply.

### 3.2. The Carnot Cycle

The idea of intrinsically irreversible processes, which is supported by kinetic simulations as well as the present macroscopic formulation and mathematical arguments, calls into question the notion of a quasi-static Carnot cycle. Figure 8 suggests that for even very small step changes in external pressure, the work of compression is greater than the work of expansion, so it is by no means clear that any cycle composed of such changes will be reversible. It is a question of exactness. Unless a cycle is exactly reversible, in as much as the same amount of work can be extracted from heat as heat from work if the cycle is reversed, the cycle cannot be said to be a Carnot cycle.

The irreversibility described in this work arises from three different mechanisms: inter-particle scattering, as represented by 0 < *α_z_* < 1; heat conduction from a reservoir, as represented by 0 < *α_h_* < 1; piston damping, owing to the dependence of the effective pressure on the speed of the piston. The key to the first two mechanisms is the idea of a delay between work performed at the piston and the consequent redistribution of energy among the degrees of freedom or the exchange of heat with a reservoir. For *α_z_* = 1, energy is instantaneously redistributed and the gas can effectively be described by the ideal gas equation of state. In consequence, the work performed on or by the gas, depending on whether the gas is expanding or being compressed, is always *PdV.* The work performed on or by the external pressure is different and the difference between the two is, by Equation (20), the kinetic energy of the piston. For *α_h_* = 0 the temperature changes with the work performed, and for *α_h_* = 1 the temperature remains constant. As shown in Figure 5, either of these conditions, in conjunction with the elimination of piston damping, leads to undamped oscillatory motion of the piston and this forms the basis of a dynamic Carnot cycle.

**Figure 9:**
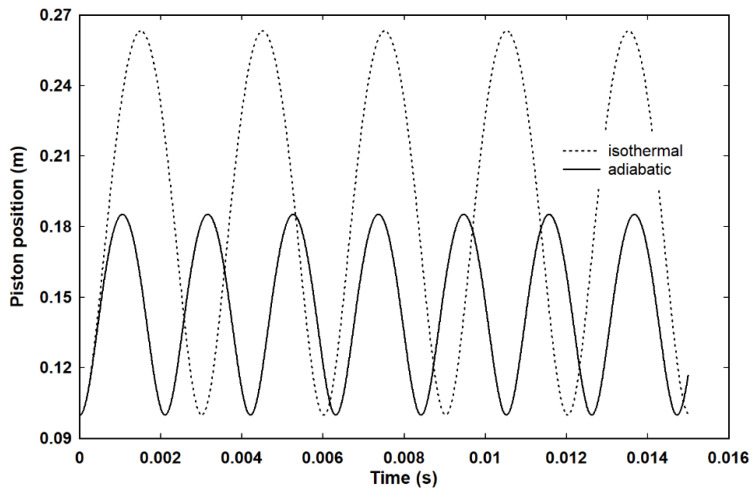
Isothermal and adiabatic undamped expansion for a gas initially at 995 K and an external pressure of two atmospheres.

**Figure 10:**
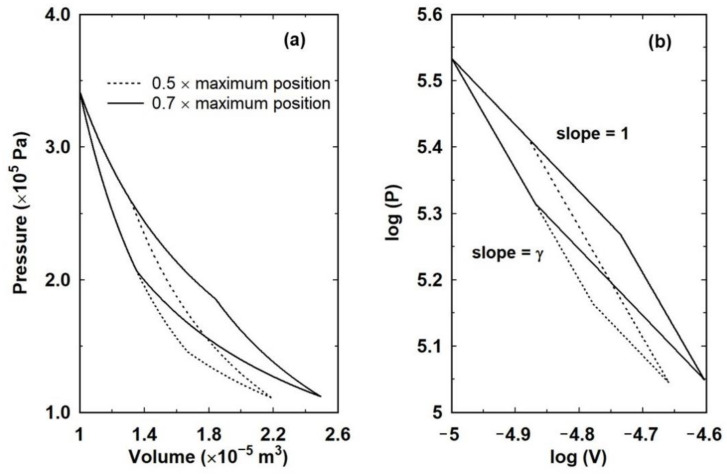
Pressure against volume for two Carnot cycles based on the isothermal expansion in Figure 8 plotted linearly (**a**) and logarithmically (**b**) to show that the curves correspond to isothermal and adiabatic transitions. Two cycles are shown to illustrate that the point at which heat flow from the reservoir is switched off is unimportant as long as the temperature of the reservoir and external pressure are adjusted at the extreme piston motion.

Figure 9 shows both isothermal and adiabatic undamped oscillations for the same ideal gas in the same cylinder, with the same starting position for the piston of 0.1 m, but at an initial temperature of 995 K acting against a constant external pressure of two atmospheres. For computational convenience, the mass of the piston still corresponds to aluminium at a thickness of 10^−4^ m, but the piston damping is idealised away by setting the ratio of *u_p_*:*u_a_* to zero for all piston speeds in simulation of a much heavier piston. It is perhaps not immediately or intuitively obvious, but an appropriate combination of ideal isothermal expansion and ideal adiabatic expansion, as illustrated in Figure 9, will cause the piston to undergo a Carnot cycle, as illustrated in Figure 10.

Figure 10 in fact shows two Carnot cycles starting from the same point: an ideal at a temperature of 995 K with the piston at an initial position of 0.1 m and an initial external pressure of two atmospheres. The initial expansion is isothermal (*α_h_* = 1) and at some point before the end point of the expansion the heat flow from the reservoir is switched off by setting *α_h_* = 0. The expansion then continues adiabatically.

The cycles in Figure 10 differ only in the point at which the heat flow from the reservoir is switched off. This affects the end point of the expansion and, therefore, the temperature of the cold reservoir, as well as the external pressure on the return stage. The precise procedure for performing the forward Carnot cycle, in which heat is converted to work, is as follows.

1. The gas is allowed to expand isothermally to some arbitrary point before the maximum of the piston motion when the heat is switched off: that is, *α_h_* is switched from a value of 1 to 0. The two cycles correspond to 0.5 and 0.7 of the maximum isothermal extent of the piston motion, which from Figure 8 corresponds to a piston position of just under 0.27 m. The temperature of the wall is set to the temperature of the gas and the total heat input during the isothermal expansion is summed. This allows for a calculation of the entropy change, $\frac{\delta Q}{T_{wall}}$. At the point when the heat flow is switched off, the piston is already in motion and the gas continues to carry out work on the piston, thereby cooling in the process.
2. At the extremum of the motion, *α_h_* is reset to 1. The temperature of the wall is set to the temperature of the gas and the external pressure is reduced. That is to say, from the entropy calculated during the isothermal stage, the nett heat that must be exchanged with the cold reservoir can be calculated. The difference between the heat in (*Q_H_*) and the heat out (*Q_C_*) must be equal to the nett work performed. If the external pressure is *P_out_* and *P_in_* on the outward and inward piston motions, respectively, and the total movement of the piston is *δx_tot_*, then

(29) $$\left|Q_{H}-Q_{C}\right|=\left|\left(P_{out}-P_{in}\right)A\delta x_{tot}\right|$$

The heat flow to the cold reservoir is summed during the isothermal compression and, at the point when the entropy change is equal to the change during the expansion stage, the heat flow is switched off by setting *α_h_* to 0.

3. The piston continues its motion, but now without any heat flow the temperature rises. The program continues until the minimum piston position is found. This turns out to be the same as the starting point and both the temperature and internal pressure are restored to their starting values.

These cycles are reversible. Starting with an adiabatic expansion and reversing exactly the steps that led to the execution of a forward Carnot cycle, one finds that the same P-V curve is mapped out.

## 4. Discussion

The remarkable feature of the dynamic Carnot cycle just described is its simplicity. It is not necessary to do anything special to force a system comprising an ideal gas to execute the cycle other than to assume the gas to be always in internal equilibrium. Then, the pressure acting on a piston is given the equation of state and the work performed by the gas is equal to *PdV*. If the external pressure is constant, representing a real physical load such as a weight being lifted, the nett force due to the difference in pressures causes the piston to accelerate. At the extremum of the piston motion, the external conditions have to be adjusted to allow the cycle to be completed. The external load has to be reduced, as represented by a lower constant pressure, and the temperature of the external reservoir has to be lowered to match the temperature of the gas at the extreme point. Not only is work performed owing to the difference in external pressures over the distance of the piston stroke but power is developed. Thus, one of the principle conceptual difficulties associated with Clausius’s interpretation of the Carnot cycle, namely that the ideal engine has to operate quasi-statically such that no power is developed, is overcome. Moreover, the cycle can be reversed simply by reversing these operations.

None of these conditions are realisable in a practical engine. A gas is not always in internal equilibrium; a piston is not always in contact with a reservoir at a constant temperature that can be adjusted to match the internal conditions at the end of the piston stroke; the heat flow from the exterior cannot be switched on or off at will; the piston stroke itself will not vary in extent if it is connected to a driving mechanism such as a cam. This makes clear the ideal nature of the Carnot cycle as a representation of an ideal engine. The formulation presented here of the processes occurring within the gas in terms of rate equations makes clear the assumptions that must be made to ensure the gas is always in internal equilibrium: instantaneous heat flow form the exterior, instant redistribution of energy within the gas, both spatially through conduction and among the different degrees of freedom through inter-particle scattering and, finally, a pressure acting on the piston which is independent of the speed of the piston.

In one sense, the existence of so many separate idealised conditions for the ideal operation of a Carnot engine is not surprising. Carnot himself assumed a similar set of steps in his own description of the operation of an ideal engine [20] (p. 18). Carnot was concerned with the development of “motive power”, but the phrase has to be understood in its historical context. The modern definition of power as the rate at which work is performed or energy exchanged was not understood at that time and instead, motive power seems to have been equated with the ability to do work. Certainly, Carnot associated motive power with expansion and regarded heat flow without expansion as a loss of motive power of heat. Therefore, he envisaged the cycle as occurring in four separate stages: expansion of a gas through heat flow from a reservoir at the same temperature as the gas, removal of the gas from contact with the reservoir and continued expansion without heat flow to change the temperature, contact with another reservoir at the same temperature as the gas and compression at a constant temperature and, finally, removal from the reservoir and continued compression back to the starting state. Reversal of the sequence of operations allowed the cycle to be reversed.

The realisation of the Carnot cycle presented in this paper is closer to Carnot’s scheme than Clausius’s. Although Carnot himself mooted the idea of reversible processes occurring in infinitesimally small steps [20] (p. 15), he did not make that a condition of the successful operation of the cycle. His focus was on a cycle consisting of the four steps above and reversing the order in which those steps are executed to reverse the cycle. This is the same notion of reversibility presented in this paper. Clausius, on the other hand, introduced the notion of a quasi-static process as reversible. Quite possibly he was influenced by Carnot, who described heat conduction between two bodies at different temperatures occurring via a series of bodies at intermediate temperatures as a way of *reversibly* maximising the motive power developed by such heat flow. He was describing a particular situation when steam, having performed work and condensed to water, needs to be returned to the state of steam. This could be performed by placing it in contact with a body at a high enough temperature, but that would result in heat flow without expansion, which is a loss of motive power, and the reverse operation of returning the heat back to the hot body would be impossible. He therefore argued that if the temperature difference between two bodies was infinitesimally small, heat could flow and do work and in reverse, work could be performed and heat would flow in the opposite direction.

Clausius gave a similar description of reversible heat flow as isothermal and, by comparison, described reversible work as occurring at the same pressure. In addition, the switch from caloric to the theory of heat as motion led Clausius to believe that large scale motions could be dissipated as heat and specifically required a reversible process to avoid “perceptible” external motion. Necessarily, this meant that the Carnot cycle must be executed quasi-statically, but it also represented a shift away from Carnot’s idea that the cycle is reversible simply by reversing the sequence of operations, to the idea that the cycle is made up of separately reversible processes. The present work shows that this need not be the case and that a reversible cycle can be executed dynamically by a piston in constant motion.

This has implications for our understanding of entropy. There are two conflicting views of entropy that need to be reconciled. The first is expressed in Equation (2), Clausius’s theorem, in which entropy is self-evidently not a state function because the sum of all changes can be less than zero round a closed cycle, and the second is that entropy is a state function because the integral of *dQ/T* between any two points in thermodynamic phase space is independent of the path. The obvious distinction between these two interpretations is that one is internal and the other external. Equation (2) defines whether a process is reversible or not with reference to the exchange of heat between a system and its environment, so in the free expansion, for example, the system can only be returned to the original state by doing work, which necessitates a flow of heat out of the system.

In contrast, the internal interpretation is not concerned with actual heat exchanges. It is primarily mathematical and is based on representing the quantities in the first law in the form of differentials. Clausius was well aware of the mathematical arguments around integrability and devoted several pages of introductory material [21] in his collection of memoirs to the topic. Born gives an account of the treatment of two variables in terms of Pfaffians and the existence of an integrating factor [22]. A small change in heat can be expressed as

(30) dQ=Xdx+Ydy

It is a property of such equations that there is an integrating factor such that

(31) dQ=λ.dϕ

The quantity *dϕ* is an exact differential and is recognizable as a change in entropy. The integrating factor, *λ*, is identified as the absolute temperature, *T*. This only applies to Pfaffians of two variables and Born shows how, for three or more variables, such that,

(32) dQ=Xdx+Ydy+Zdz

a geometric interpretation can be attached to this in the form of a set of planes perpendicular to the surfaces in which *ϕ* is constant. Dunning-Davies also gives an account of the mathematical interpretation of entropy in terms of level surfaces within thermodynamic phase space [23]. States lying on the same surface can only be accessed by adiabatic transformations such that *Dw = PdV*. If *dW ≠ PdV,* or if *dQ* ≠ 0, the transformations occur between states on different surfaces, with the former requiring a transition to a surface representing a higher value of entropy.

Entropy, thus defined, is a consequence of the differential geometric properties of thermodynamic phase space, and the question arises as to whether there is a corresponding physical property of a system in that same state. Clausius certainly believed so. It was common at the time to consider heat as a property of a body, in as much as a body was believed to contain a certain amount of heat at a certain temperature. It would follow from such a belief that any change *δQ/T* is changing *some* property of the body. As we have seen from Maxwell [14], however, there was a growing shift away from the notion of heat as motion, or kinetic energy, to the idea that heat is associated with an exchange of energy in some form and this is clearly the most appropriate interpretation of Equation (2). Nevertheless, entropy is still regarded as a property of a body today, but it is the very idea of entropy as a physical property, with the units of joules per degree Kelvin, that leads to a conflict with energy conservation.

This conflict can be traced directly back to Clausius. He appears to have retained the belief that heat was a property of a body in the sense given above well into the second half of the nineteenth century, and quite possibly until his death. He also believed that one of the principal effects of heat was to cause a separation between the constituent particles. He called this the separative force of heat and attempted to express this mathematically through the change in disgregation, *dZ*. This quantity replaced work performed in what is essentially a mathematical expression for the conservation of energy, in order to derive an expression identical to Equation (2). It should be noted that in modern usage the inequalities have different signs but, originally, Clausius defined a positive *dQ* in such a way as to make the integral in Equation (2) positive or zero and it appeared in this form in his earlier works. It was only later, in the Ninth Memoir, that he changed the sign of *dQ* to correspond with the modern view that a negative *dQ* equates to a loss of heat by the system. As derived by Clausius, the inequalities were identical and it was this later change of sign that made them different.

Clausius’s thinking behind these inequalities is based on something he referred to as “uncompensated” transformations. Clausius’s whole theory of the thermodynamics of cyclic processes was based on what he called the equivalence of transformations. The concept is difficult to understand, being based in part on Clausius’s outmoded view of heat, and has disappeared from the modern thermodynamic lexicon. However, for the purposes of this discussion, we can focus on the transformation of heat into work and vice versa, work into heat. Clausius assigned such transformations an equivalence value (*aequivalenzwerth* in German) of *δQ/T*, where *δQ* is a change in heat, and in a cyclic process such as the Carnot cycle, the equivalence value of the isothermal expansion is compensated by an equivalence value of the isothermal compression. In an irreversible cycle, for example, if the isothermal expansion is replaced by the free expansion, one of the transformations will be uncompensated. There will be a negative value of *δQ/T* around the cycle, corresponding to a net outflow of heat. Clausius sought an equivalent uncompensated transformation for a single, irreversible, non-cyclic process and focused on work. By setting *TdZ ≥ dW* in Equation (4), he arrived at the sought-after inequality. In a reversible process, *TdZ = dW* and *dQ/T* are compensated by the combined effect of the changes in both *dZ* (=*dW/T*) and *dH/T*, or their integrals over larger changes, but for an irreversible process, *TdZ > dW* and *dQ/T* remain uncompensated.

Clausius believed the equivalence value to be a property of a body and it was quite natural for him to extend that to the entropy, but therein lies the origin of the conflict with energy conservation identified in the introduction. Whilst it might well be true that the work performed in a so-called “reversible” process is always algebraically greater than the work performed in an irreversible process, the total change in internal energy for a system containing a fixed number of particles is given by the sum of heat exchanged and work performed, not the work that could be performed in a different process. By associating the work that can be performed, as opposed to the work that is performed, with a physical property of a body, Clausius derived a quantity that does not adhere to energy conservation if the work performed is less than the so-called irreversible work.

This explains why Clausius believed entropy to be the property of a body, but it does not explain why later thermodynamicists continued to accept this view in light of the emergence of the concept of energy. Undoubtedly, the idea of entropy as a state function played a part. Clausius clearly regarded his own mathematical arguments on integrability to provide a mathematical explanation for his interpretation, and as the argument against entropy being a property of a body is physical rather than mathematical, later mathematical treatments would not have provided any kind of counter argument. For example, Boyling [24,25] asserted that entropy exists based on purely mathematical arguments. He considered the space of equilibrium states as a differentiable, topological manifold, the properties of which are determined through the existence of differential functions representing work and heat and internal energy that links the states. A global integrating factor exists that is the inverse of temperature, and he concludes, therefore, that entropy exists. It seems unlikely that Boyling was simply re-stating a mathematical truth. More likely, he was suggesting that the existence of a global integrating factor, coupled with the physical nature of heat, work and internal energy as the quantities involved, means that entropy is a physical property of thermodynamic states.

For entropy to be a property of a body and to increase in irreversible processes, there has to exist a reversible process in which work performed in either direction is given by *PdV.* The nature of reversible processes has long exercised thermodynamicists and recently Norton has summarized the difficulties [26]. Quite possibly, there is no such thing as a quasi-static, reversible process [16]. The assumption underlying the work presented in this paper is that a step change in external pressure is required to bring about a change in state. The existence of internal damping means that the ensuing work is intrinsically irreversible: that is, the work of compression will be greater than the work of expansion. As Figure 8a makes clear, there is no sense that *PdV* approaches a limiting value that is effectively the same in both directions and, no matter how small the change, some heat will have to be extracted to restore the initial state. By Equation (2), such processes are irreversible.

The conventional interpretation of the need to extract heat to return the system to its original state means that the entropy of the gas has increased during the irreversible process. However, the argument that this increase in entropy represents a real, physical property of a gas in the state represented on the *P-V* diagram rests on the possibility, even if it is difficult to realise in practice, that there exists a reversible path between the two states. Boyling’s definition of reversible is equivalent to a point representing a thermodynamic state moving in the manifold. The existence of two different slopes in Figure 8b shows that even for small step changes in external pressure, motion along adiabats is not possible. The cause can be traced to the need to damp the piston motion. Unless the kinetic energy of the piston is dissipated, the piston will simply oscillate. The existence of internal, and therefore intrinsic, mechanisms of damping means that continuous, and therefore integrable, motion of a physical system along isotherms and adiabats would appear to be impossible. Therefore, the idea of a reversible process represented by work *PdV* would appear to be a mathematical abstraction.

This brings us to the Carnot cycle as executed in this work. It is a dynamic cycle executed over the expansion and return stroke of a piston that is always in motion. The conditions and assumptions necessary for the cycle to be realised mean that, at any given instant, the gas has an identifiable temperature and pressure. Although the gas never occupies a static equilibrium state, hence the term “dynamic cycle”, the continuous motion of the piston means, in effect, that the thermodynamic state of the gas, as defined by the pressure, volume and temperature, changes continuously. In effect, the state of the gas can be considered as a point moving along isotherms or adiabats, depending on the heat flow from the reservoir. As such, the expansion and compression strokes are reversible. As Figure 9 shows, under the idealised conditions of no damping, both isothermal and adiabatic expansions are cyclic, which is to say that on its return the piston follows exactly the reverse trajectory through thermodynamic phase space. Even if the heat flow from the reservoir is switched off at some point during the piston stroke so that the expansion changes from isothermal to adiabatic, as long as the external conditions remain unchanged and provided the heat flow is switched back on at the same point, the piston will reverse its path through thermodynamic phase space. However, if the external pressure and temperature of the reservoir are adjusted at the end of the piston stroke, the gas can be made to follow a different trajectory back through thermodynamic phase space. It follows that in mapping a continuous motion through thermodynamic phase space, the entropy change associated with heat flow to or from the reservoir must match the mathematical property of entropy associated with the states in that phase space. Therefore, if the entropy change on the return stroke matches the entropy change on the outward stroke, a dynamic Carnot cycle can be executed.

## 5. Conclusions

Some of the conceptual difficulties around entropy have been discussed to illustrate its confusing and contradictory nature. In particular, attention has been drawn to the idea that an increase in entropy means that some property with the units of energy, *TdS*, is increasing in a way that is incompatible with energy conservation. The origin of this difficulty has been traced back to Clausius, who actively sought an inequality similar to that he had developed for cyclic processes in which the maximum work is performed in a reversible process. It has been shown that he based his development of the concept of reversibility not on conservation of energy but on the idea that a body at a given temperature contained an identifiable amount of heat and that this heat had associated with it a force that could do work. He justified the inequality for a single, irreversible process through the inclusion of reversible work in his mathematical development of entropy rather than the work that is actually performed, and thereby built into the concept of entropy the potential for a quantity with the units of energy to increase outside the bounds of the first law. The argument is succinctly summarized in Equation (10), which shows how the entropy increase in an irreversible process depends on the difference between the work that is actually performed and the work that *could* be performed in a corresponding reversible change between the same two states.

It has also been argued that the idea of reversible work is a mathematical abstraction. Physically, a step change in external pressure is required to bring about a change of state and the presence of internal damping means that the ensuing work is intrinsically irreversible. However, the same states can be represented as points in thermodynamic phase space and an integrable path between can be defined through differential geometry with the work performed being expressed in the form *PdV*. This is a necessary condition for an integrating factor, and therefore the state function called entropy, to exist. In contrast, in the intrinsically irreversible processes described in this paper, the work performed between any two states is not expressible in terms of state variables and no integrating factor exists for such transitions. Nonetheless, entropy can be said to exist as a consequence of applying the techniques of differential geometry to the graphical representation of thermodynamic states in thermodynamic phase space. The question arises as to whether the mathematical state function is also a physical property of a body and grounds for thinking that these are not the same have been presented in this paper.

The microscopic scattering processes underlying the internal damping mechanisms has been formulated macroscopically using rate equations that couple energy changes in the gas to work performed at the piston. The irreversible nature of work performed following a step change in external pressure has been demonstrated through numerical simulations. It has also been shown that in the ideal case when these damping mechanisms are assumed not to operate, the gas behaves as if it is in internal equilibrium at all times. The pressure acting on the piston is equivalent to the equilibrium pressure and the gas does work *PdV.* The difference between the work performed internally and externally, against the constant load, becomes kinetic energy in the piston. As the piston moves, the system maps out a trajectory through the equilibrium states of thermodynamic phase space. The change in entropy of the states corresponds exactly to the change in entropy due to the exchange of heat with the reservoir and, with appropriate adjustments to the temperature of the reservoir and the external pressure, the gas inside a piston can be made to undergo a Carnot cycle.

This dynamic cycle is a conceptually less challenging alternative to the quasi-static Carnot cycle in several respects.

- Although the quasi-static approach to reversible processes is accepted as standard, it is not compatible with the idea that a difference of pressure must exist in order to change the state.
- In the dynamic cycle, on the other hand, the external pressure is different from, and independent of, the internal pressure and the movement of the piston is a consequence of simple mechanical principles.
- The Carnot cycle is intended to represent the ideal engine and the assumption of ideal conditions within the gas to eliminate damping mechanisms is fully compatible with that.
- There is no need to consider whether entropy is a property of a body or not as the entropy change due to the heat flow from the reservoir necessarily corresponds to the state function of thermodynamic phase space.
- Finally, the absence of damping means that the system can map out the reverse trajectory in thermodynamic phase space under the same external conditions and, in consequence, the cycle itself is reversible.

## Data Availability

The system modelled in this paper is illustrative and the dimensions chosen arbitrarily. The data was generated by simple, purpose-written python code and can easily be reproduced. Should the specific data be required, it can be obtained on request to the author.
